# Immune Dysfunction in HIV: A Possible Role for Pro- and Anti-Inflammatory Cytokines in HIV Staging

**DOI:** 10.1155/2017/4128398

**Published:** 2017-11-02

**Authors:** Iorhen Ephraim Akase, Bolanle O. P. Musa, Reginald Onyedumarakwe Obiako, Abdurrahman Ahmad Elfulatiy, Abdullahi Asara Mohammed

**Affiliations:** ^1^Department of Medicine, Ahmadu Bello University Teaching Hospital, Shika-Zaria, Nigeria; ^2^Immunology Unit, Ahmadu Bello University, Zaria, Nigeria; ^3^Neurology Unit, Ahmadu Bello University, Zaria, Nigeria

## Abstract

HIV infection is a chronic infection that almost inevitably progresses to AIDS. The infection is characterized by the deterioration in the immune function leading to opportunistic infections and malignancies. Additionally, there is an associated immune dysfunction characterized by a persistent inflammatory state and unhealthy elaboration of both pro- and anti-inflammatory cytokines. The CD4^+^ T cell count has been used as a surrogate for the level of immune dysfunction that exists in patients with HIV infection. Eighty-eight (88) patients with HIV infection, forty-four (44) of whom were treatment naïve patients and forty-four (44) who were treatment-experienced patients, were recruited. The serum concentrations of cytokines IL-6 and IL-10 were carried out using R&D human *Quantikine* ELISA kits, while patients' CD4^+^ T cell counts were evaluated using the *Partec easy count kit*. The serum IL-6 and IL-10 concentrations were significantly higher among the AR-naïve participants compared to the ART-experienced group. Additionally, the IL-6 and IL-10 concentrations were higher in patients with lower CD4^+^ T cell count compared to those with higher cell counts though this was not statistically significant. Also, both IL-6 and IL-10 concentrations were higher in patients with higher WHO clinical staging of disease, significantly so for IL-6.

## 1. Introduction

The human immunodeficiency virus type-1 (HIV-1) infection and its sequelae, the acquired immune deficiency syndrome (AIDS), are the major causes of morbidity and mortality worldwide, accounting for over 35 million deaths since the first reported cases in 1983 [1]. AIDS is characterized by various abnormalities of immune function which manifests as opportunistic diseases that eventually lead to increased morbidity and mortality in involved individuals [2]. An estimated 36.7 million people are infected with HIV worldwide with 69.5% of this number living in sub-Saharan Africa [1]. In Nigeria, about 3.1% of the population are said to be infected with HIV [1].

Cytokines are a group of low molecular weight proteins that mediate communication between immune system cells. They contribute to chemical signaling pathways that regulate cell development, tissue repair, haematopoiesis, inflammation, and immune responses. They act in a complex network where one cytokine can influence the production of, and response to, many other cytokines [3]. Cytokines play a crucial role in entry of HIV into host tissues, progression of disease, and in the occurrence of opportunistic infections [4]. HIV infection is characterized by immune activation, with the production of both pro- and anti-inflammatory cytokines. An immune deficiency state results when HIV infection produces a significant decrease in number of CD4^+^ T lymphocytes, defective function of both the T lymphocytes and macrophages, and dysregulation of cytokine production [5].

The CD4 T lymphocytes, also called T helper cells, are important in the coordination of the immune system by directing immune responses against pathogens either towards a predominantly humoral or a cell-mediated response depending on the nature of the antigen and the immune receptor that has been activated by the antigen. Additionally, subsets of the T helper cells are crucial in the regulation of immune responses in immunocompetent individuals. By selectively targeting the CD4^+^ T cell, infection with HIV not only renders an immunodeficient individual but also produces a state of immune disarray which manifests as a clinical predisposition to infections and a persistent inflammatory state which has been demonstrated even in individuals who are on cART and who have had virological suppression with viral suppression below detected serum levels.

Improvement of patients with HIV is documented clinically by the improvements in clinical conditions, virologically by the reductions in HIV RNA viral loads, and immunologically by the CD4^+^ T cell count. For long, clinical decisions in HIV-infected individuals have been based on the CD4^+^ T cell counts of such individuals, but questions remain as to how well the peripheral CD4^+^ T cell count mirrors the true state of the immune dysfunction seen in patients who have been infected with HIV.

The aim of this study was therefore to assess the relationship between the CD4^+^ T cell count and the serum levels of IL-6 and IL-10 in patients infected with HIV whether on treatment or not, who were attending the ART clinic of the Ahmadu Bello University Teaching Hospital, Shika-Zaria.

## 2. Methods

This study was carried out in the outpatient clinic of the Ahmadu Bello University Teaching Hospital (ABUTH) HIV program. The HIV program is situated in the NASARA complex and is part of the 700-bed referral hospital located in Shika-Zaria, Kaduna State. Eighty-eight (88) patients were selected consecutively. They included forty-four (44) newly diagnosed ART- (antiretroviral treatment-) naïve patients to comprise the test group and another forty-four (44) ART-experienced patients to serve as the comparison group. The comparison group included patients who had been receiving ARTs for at least 6 months. All eighty-eight samples (88) had IL-6 and IL-10 assays carried out on them, as well as CD4^+^ T cell enumeration.

The study was a comparative cross-sectional study. Ethical approval for the study was obtained from the ABUTH Ethical and Scientific committee. Adequate counseling and informed consent were applied confidentially throughout the course of the research. Data was collected using a researcher-administered questionnaire, which captured the biodata/sociodemographic characteristics, medical history, and the physical examination of the participants.

The levels of both serum IL-6 and IL-10 were assayed using the *Quantikine*® ELISA human IL-6 and IL-10 immunoassay kits by R&D Systems Inc., USA. The CD4^+^ T cell estimation was done with the *Partec Cyflow* flow cytometer using the CD4 *easy count kit* (Sysmex Partec GmbH, Germany).

All data was analyzed using Statistical Package for the Social Sciences (SPSS), version 20.0, Chicago, IL, USA. Qualitative variables were reported as percentages. Normally distributed quantitative variables (e.g., age) were presented as mean and standard deviation, while those with uneven distribution (e.g., CD4 count and cytokine levels) were presented as median and range. Statistical test of significance was set at 5% alpha level using the chi-square and Pearson's correlation for qualitative and quantitative variables, respectively. The Student *t*-test was used to compare the mean age while the Mann–Whitney *U* test was used to compare the medians of CD4 cells, interleukin-6, and interleukin-10. Linear regression model was used to measure the relationship between the interleukin levels and the WHO clinical stage, antiretroviral (ARV) regimens, and ARV treatment categories.

## 3. Results

### 3.1. Sociodemographic Characteristics

Males constituted 54.5% (48) of the study participants while 45.5% (40) were female. The mean age in the study was 36.6 ± 8.8 years, and the majority of the respondents were of the Hausa/Fulani extraction, constituting 51% (45) of the participants. Participants of Ibo and Yoruba extraction each constituted 4.5% (4) of the respondents.

### 3.2. Immunological Parameters

The median serum interleukin-6 concentration was lower among the female participants compared to the male counterparts, with median values of 3.74 pg/mL (0.00–171.00 pg/mL) and 2.36 pg/mL (0.00–45.31 pg/mL) among the male and female participants, respectively (*p* = 0.88, Mann–Whitney *U* test). Similarly, the median serum concentration of interleukin-10 was lower among the female participants compared to the male participants, with median values of 6.76 pg/mL (0.00–134.20 pg/mL) and 7.22 pg/mL (0.38–47.37 pg/mL) among the female and the male participants, respectively (*p* = 0.68, Mann–Whitney *U* test).

The median CD4^+^ T cell count in the ART-naïve group was 166 cells/*μ*L with a range of 6–809 cells/*μ*L, while among the ART-experienced participants, the median was 463 cells/*μ*L with a range of 37–1519 cells/*μ*L (*p* < 0.001, Mann–Whitney *U* test). The median value of the serum concentration of IL-6 among the ART naïve group was 6.8 pg/mL with a range of 1.2–316.0 pg/mL. Conversely, the median serum IL-6 concentration among the ART-experienced subjects was 1.40 pg/mL with a range of 1.0–27.5 pg/mL (*p* < 0.001, Mann–Whitney *U* test). Similarly, the median IL-10 concentration among the ART-naïve subjects was 10.1 pg/mL with a range of 2.1–870 pg/mL, while a median value of 6.0 pg/mL and a range of 3–39.9 pg/mL (*p* = 0.14, Mann–Whitney *U* test) was observed among the ART-experienced subjects (see Table 1).

The association between the patient categories (i.e., ART naïve versus ART experienced) and serum interleukin-6 levels was not affected after adjustment for the gender of the participants, with the regression coefficient for patient category reducing by 0.13% (*b =* 15.76 versus *b =* 15.74). Similarly, the effect of the participant gender on the difference in the serum interleukin-10 among the study participants was not significant, with the regression coefficient only reducing by 3.33% after adjustment for gender (*b =* −1.80 versus *b = −*0.06).

Both the serum IL-6 (*r* = −0.43, *p* = 0.01) and IL-10 (*r* = −0.27, *p* = 0.12) concentrations showed an inverse correlation with the CD4^+^ T cell count of the participants in the study participants. The serum concentrations of both interleukin-6 and interleukin-10 demonstrated a positive relationship with the WHO clinical stage of participants (see Table 2).

There was no significant increase of interleukin-6 levels with the WHO clinical stage II of HIV compared to stage I (*b =* 3.98, *t*(83) = 0.51, and *p* = 0.62); however, the increase in serum interleukin-6 levels was significant at stage III (*b =* 20.27, *t*(83) = 2.31, and *p* = 0.02) and stage IV (*b =* 141.64, *t*(83) = 9.66, and *p* < 0.001) compared to levels at WHO clinical stage I, with the regression model predicting 53.5% (*R*^2^ = 0.535) of the variance (*F*_(3, 52)_ = 32.24, *p* < 0.001). There was also a nonsignificant increase in the serum interleukin-10 levels at WHO clinical stage II (*b =* 15.56, *t*(52) = 0.55, and *p* = 0.56) and stage III (*b =* 23.06, *t*(52) = 0.77, and *p* = 0.44) compared to stage I; however, the increase in interleukin-10 was significant in stage IV compared to stage I (*b =* 301.34, *t*(52) = 6.06, and *p* < 0.001), with the regression model predicting 41.7% (*r*^2^ = 0.417) of the variance (*F*_(3, 84)_ = 12.40, *p* < 0.001).

### 3.3. Interleukin Levels and HAART

As shown above, there was a significant difference in the median serum concentrations of IL-6 among the study categories of patients, with levels significantly higher among the treatment-naïve subjects compared to the ART-experienced subjects (*p* < 0.001, Mann–Whitney *U* test).

The IL-6 levels were affected by the ARV regimen the patient was currently taking, with the highest levels found in participants who were not on any medications and the lowest levels among those who were taking Tenofovir/Lamivudine/Efavirenz combination (1.45 pg/mL, *b =* −22.00, *t*(82) = −1.78, and *p* = 0.08), while participants who were on Zidovudine/Lamivudine/Nevirapine combination had higher levels among those who were on the other regimens (2.29 pg/mL, *b =* −14.53, *t*(82) = −1.88, and *p* = 0.06) compared to those who were yet to commence treatment, with the ARV regimen explaining only 8% of the variance in the interleukin-6 levels (*r*^2^ = 0.08, *F*_(5, 81)_ = 1.41, and *p* = 0.23). Participants who were on Tenofovir/Emtricitabine/Efavirenz combination had the lowest median serum interleukin-10 concentrations (9.71 pg/dL, *b =* −45.58, *t*(50) = −1.19, and *p* = 0.24) with the highest serum concentrations of interleukin-10 in the ART-experienced group observed among patients who were on Tenofovir/Lamivudine/Lopinavir-ritonavir regimen (15.8 pg/mL, *b =* −43.04, *t*(50) = −0.67, and *p* = 0.51) compared to the ART-naïve group, with the ARV regimen explaining 4.1% of the variance (*r*^2^ = 0.041, *F*_(5, 50)_ = 0.43, and *p* = 0.83).

The levels of IL-6 were affected by the stage of treatment, with the highest levels detected among those yet to commence medications, followed by those on the 2nd line drugs (2.22 pg/mL, *b =* −19.15, *t*(84) = −1.83, and *p* = 0.07) while the lowest levels were detected among those on the 1st line medications (1.80 pg/mL, *b =* −16.03, *t*(84) = −2.34, and *p* = 0.02). The stage of ARV treatment explained 7.6% of the variation (*r*^2^ = 0.076, *F*_(2, 84)_ = 3.46, and *p* = 0.04). The IL-10 levels were also noticed to be higher among the untreated participants but lower among the participants who were on the 1st (11.65 pg/mL, *b =* −45.38, *t*(53) = −1.50, and *p* = 0.14) and 2nd (12.59 pg/mL, *b =* −47.41, *t*(53) = −1.02, and *p* = 0.31), with the regression model predicting 47% of the variation (*r*^2^ = 0.47, *F*_(2, 53)_ = 1.31, and *p* = 0.28). The distribution of the participants according to the stage of treatment is shown in Table 3.

There was a significant negative correlation between the duration from diagnosis to time of study and the levels of interleukin-6 (*r* = −0.25, *p* = 0.03). The duration from diagnosis of HIV infection to time of study demonstrated a nonsignificant negative correlation with the serum interleukin-10 (*r* = −0.19, *p* = 0.18).

## 4. Discussion

This study showed higher interleukin levels among patients with lower CD4^+^ T cell counts, which is consistent with more pronounced inflammation in subjects with more advanced disease. These findings are also consistent with similar studies done elsewhere [6, 7]. Similarly, the higher serum concentrations of both IL-6 and IL-10 in patients with advanced WHO clinical stage of disease mirrored the pattern observed with low CD4^+^ T cell counts and corresponding high interleukin levels. These findings add credence to the usefulness of the WHO clinical staging in decision-making, especially in resource poor settings that do not have access to routine CD4^+^ T cell measurements. The higher serum levels of both IL-6 and IL-10 in ART naïve compared to the ART-experienced HIV-positive participant categories are also consistent with studies that show higher degrees of inflammation with deteriorating immune function [6–9], in line with the thinking that the HIV virus drives the inflammation that is persistent in HIV infections [2]. Similarly, the improvement that is associated with the use of ARTs as evidenced by immunological improvement in the patients has been observed by other researchers [10, 11]. It is also worthy of note that the ART-experienced patients also have persistently elevated levels of inflammatory markers albeit in lower concentrations than in the ART-naïve participants. This is consistent with the findings by other researchers that, despite successful suppression of viral replication to undetected levels, a degree of inflammation persists, which is vital to the overall pathogenesis of HIV, which may be of consequence in the long-term outcomes of these patients [2, 12].

The findings in this study suggest that participants on Tenofovir/Emtricitabine/Efavirenz and Tenofovir/Lamivudine/Efavirenz combinations had lower levels of both interleukin-6 and interleukin-10, with higher levels observed among participants who were on Zidovudine/Lamivudine/Nevirapine combination and the protease inhibitor-containing regimens. This is in contrast to findings by workers elsewhere that suggest the suppression of interleukin levels was without respect to the ART regimen used [11, 13]. The methodology of this study however is insufficient to adequately address this issue, and a well-designed randomized clinical trial will be better suited to evaluate this.

Patients who had a longer duration between diagnosis and the conduct of this study were observed to have lower interleukin levels. Whether this observation is due to a more sustained viral suppression is uncertain as we did not assay for HIV viral load in our participants.

## 5. Conclusion

There are higher concentrations of IL-6 and IL-10 levels among HIV patients that are not on HAART, and the levels of IL-6 and IL-10 are reduced by HAART. There is a higher level of IL-6 and IL-10 among HIV patients with low CD4^+^ T cell counts and advanced WHO clinical stage, which was not affected by the gender of the participants. This relationship between interleukin-6 and interleukin-10 suggests that there may be a role for the use of cytokine measurements in the staging of HIV infections and this may be very useful in the setting of poor immune reconstitution with poor CD4 T lymphocyte response which is not uncommon in resource poor settings, partly on account of a severely damaged lymphoid system due to late commencement of ARVs.

## Figures and Tables

**Table 1:** Immunological parameters of participants.

Parameter	ART naïve	ART experienced	*p* value^∗^
Median^†^ CD4 concentration	166 cells/*μ*L (6–609)	463 cells/*μ*L (37–1519)	<*0.001*
Median IL-6 concentration	6.8 pg/mL (1.2–316)	1.4 pg/mL (1.0–27.5)	<*0.001*
Median IL-10 concentration	10.1 pg/mL (2.1–870)	6.0 pg/mL (3.0–39.9)	*0.14*

^∗^ *α* < 0.05, Mann–Whitney *U* test. ^†^Median (range).

**Table 2:** Median concentration of interleukins according to the WHO clinical stage of HIV.

WHO clinical stage	IL-6 concentration (pg/mL)	IL-10 concentration (pg/mL)
1	2.22 (0.00–37.97)^∗^	10.15 (0.00–47.37)
2	2.22 (0.00–45.31)	26.32 (5.64–43.23)
3	7.76 (1.39–65.13)	30.83 (12.40–48.50)
4	171.00 (4.70–175.84)	134.20 (14.66–800.00)

^∗^Median (range).

**Table 3:** Antiretroviral stage of treatment based on the sex of the participants.

ARV category	Male	Female	Total
ART naïve	26	18	44
1st line regimen	18	16	34
2nd line regimen	4	6	10
